# La Roche on Yellow Fever

**Published:** 1853-04

**Authors:** 


### LA ROCHE ON YELLOW FEVER.

For a number of years past Dr. La Roche, of Philadelphia, has been engaged in the collection of materials for a work on Yellow Fever, in which he meant to give the history of the various irruptions of the epidemic in this country. Such a work, prepared with the industry, judgment, and fidelity which Dr. La Roche is sure to bring to the task, could not help proving an acceptable and valuable addition to our medical literature. We feel a greater desire to see the work brought to completion since reading some papers on yellow fever, from the pen of the author, in the Charleston Medical Journal, and the American Journal of the Medical Sciences. The labor bestowed by Dr. L. on the subject is evidently very great. He has posseesed himself of all the important facts connected with it. Among other things, he has convinced himself that the disease is not contagious under any circumstances, but originates always in local sources of miasmatic infection. Ships having the infection on board may introduce it into a place, but never a clean vessel with a healthy crew, from whatever port it may have sailed; and hence the futility, in Dr. La Roche's view, of those quarantine regulations which detain vessels free from the infection, because they have chanced to come from infected points. We trust we shall soon have Dr. L's. work on this interesting subject in extenso.

